# Encephaloid Cancer of the Liver in a Child

**Published:** 1877-04

**Authors:** C. J. Lewis


### ENCEPHALOID CANCER OF THE LIVER IN A CHILD.

By DR. C. J. LEWIS.

John A. Aet. 13; born in Sweden; came under my care Aug. 20, 1876. In April last this boy was kicked by an older brother in the right side, just below the short ribs, causing him to remain in the house for about a month. He was visited by a physician twice for this injury, and he so far recovered as to be able to attend school for a few weeks. There was slight ecchymosis at seat of the injury, a little general fever, and some tympanitis. About two weeks after the hurt, looseness of the bowels supervened, accompanied by vomiting. In the early part of July an eruption appeared on the face and back of the hands, unaccompanied with itching. His appetite was small from an early period of his illness; but he continued thirsty. During the latter part of July, it was observed that his abdomen began to swell.

I attended this lad through measles in July and August, 1875, he making a good recovery. He had been thinly clad and improperly and scantily nourished. *No history of cancer in the family.*

On examination, I found the skin of normal temperature, dry, having on the face, arms, upper part of the chest and shoulders a fine and quite closely studded papulous eruption of a yellowish brown color, and coming in crops. The abdominal subcutaneous veins were very much enlarged. The pulse 105, small, compressible; the central portion of the tongue was coated with a brownish fur—tip and edges smooth and red; the bowels moving from eight to twelve times daily, mostly at night; urine scanty and of high color; appetite small, with some thirst. He was in bed—decubitus dorsal, with his lower extremities extended; was small in stature, having a careworn expression. His sleep was broken by his frequent stools, which at times were watery, light in color, at other times dark and grumous. There was a tumour filling the abdomen from the navel upward into the chest, and extending on the right side down to the pelvis. It was not tender nor adherent to the abdominal wall, but firm and immovable on under side. This was regarded as an enlarged liver. From the coppery color of the eruption, together with the scaly desquamation of the epidermis, the tumour was looked upon as having a syphilitic origin.

Aug. 22d. Has been easier since my first visit than for several weeks; vomits but little; the diarrhœa is lessened; skin moist; tongue is cleaning; pulse 85, with no perceptible change in the tumour.

Aug. 26th. Has taken no medicine for nearly two days, and for about a day and a half, the stools have been frequent, the vomiting urgent; pulse 120, very small; has had but three hours sleep in two days. I now suspect cancer of the liver.

Sept. 7th. Since the above report, he has not suffered much from pain; the stools—three to five daily—have generally been of a light brown color, although occasionally one would be like dark coffee-grounds; his sleep has been fair; pulse 84; but the tumour is evidently enlarging, and having now some liquid effusion in the abdominal cavity. Dr. J. R. Buchan saw the patient this time with me, and he also regards the disease as malignant.

Sept. 10th. The patient has taken but a few doses of medicine since the 7th inst., alleging as a reason that it "burned his stomach." Since the omission of the opium, the pain has been more or less severe all the time, with occasional spells of excruciating pain. I now ordered grain doses of powdered opium at such intervals as to allay pain.

Sept. 16th. He has been kept under the influence of opium since the 10th inst., and is so now. Whenever the effects of the opium were allowed to pass off, the pain would return, vomiting would soon ensue, with frequent stools. His pulse is 112 per minute, and a mere thread. His appetite has failed and he is rapidly sinking.

He at no time evinced much tenderness in the tumour on pressure. The emaciation was extreme. Severe or more or less constant pain was complained of, only during the last two weeks of his illness. Death relieved him on the 19th inst.

Autopsy 23 hours after death.

I was assisted in the autopsy by Drs. A. B. Strong, J. R. Buchan and F. B. Eisen Bockius. The organs in the abdominal cavity were examined and found in a normal condition, excepting the liver—which specimen is here presented. The liver was somewhat nodulated and doughy to the feel. The anterior surface presented a mass of yellowish white granular tissue, having but little of normal liver appearance, and the mass occupied nearly all of the abdominal cavity. The right lobe extended to the left of the median line, strongly pushing the pancreas against the spinal column, and terminating in a heart-shaped cyst. The left lobe extended well down into the pelvic cavity. The weight of the liver is six pounds and ten ounces, (avoird.). It was adherent to the underlying tissue by ligamentous bands along its entire dorsal side. To the naked eye, the posterior surface had no encephaloid granules, excepting the three cysts:—two under the right lobe and one under the left. These cysts were round or oblong, and about 2½ in. in diameter, containing fluid and adherent to the liver. The gall-bladder was distended with a thick, dark fluid. The gastric, pancreatic and renal veins were greatly enlarged and full of dark blood; so also were the vessels supplying the structure of the liver. The appearance of the liver on section was similar to that presented by fresh suet. A specimen was left with Dr. Danforth for microscopic investigation.

*Microscopy.*—The specimen left with me presented very clearly the usual appearances of encephaloid cancer. A luxuriant cell growth, presenting an almost endless variety of cells, as regards form and dimensions—in fact, the "atypical" cells of the malignant new formations;—together with the usual number of fat globules, fatty granules and degenerating hepatic cells, which gives a very complete picture of encephaloid cancer as it commonly occurs in the liver.

Two points in the case seem to me especially worthy of attention. *First:* the fact that the liver was the only organ or part invaded by the disease. It is comparatively rare that we find encephaloid cancer confined to any one part or organ, simply on account of the facility with which it is disseminated. "In some instances, the liver is the only organ infected with cancer, or is the organ *in which the cancer originated;* but far oftener the formation of cancerous tumors in it, is consequent on cancer of some other part, especially the stomach and the breast." (Budd, "Diseases of the Liver," p. 394.) On the other hand, Rokitansky says, "Carcinoma of the liver is a disease of much greater importance, than tubercular depositions, as it occurs very frequently and is often a primary affection. (Path. Anat. II., p. 121.) This seems to imply that hepatic cancer is by no means a rare disease in Rokitansky's experience, but such is not the experience of pathologists in this country. A second point of interest is the age of the patient. I think it will be generally agreed that primary cancer of the liver—or indeed of any organ—is rarely seen in childhood.

